# From Anxiety to Contentment: The Role of Multiple Mediations and Privacy Concerns in the Transition from the FOMO to the JOMO Among Dating App Users

**DOI:** 10.3390/bs15020168

**Published:** 2025-02-04

**Authors:** Yuanhao Li, EunKyoung Han

**Affiliations:** Department of Media and Communication, Sungkyunkwan University, Seoul 03063, Republic of Korea; rocca95@g.skku.edu

**Keywords:** FOMO, JOMO, self-disclosure, social media stalking, user fatigue, privacy concerns

## Abstract

This research explores the psychological transition that occurs in dating app users from the Fear of Missing Out (FOMO) to the Joy of Missing Out (JOMO) using the Stressor–Strain–Outcome (SSO) model. An online survey of 410 Tinder users reveals that the FOMO significantly influences self-disclosure and social media stalking behaviors, which leads to user fatigue and eventually the JOMO. This survey also finds that privacy concerns play a moderating role in this process. In particular, the results show that a heightened FOMO increases self-disclosure and social media stalking, which intensifies fatigue and fosters the JOMO. Privacy concerns significantly modulate the relationship between the FOMO, fatigue, and the JOMO, thus playing a critical role in user interactions with dating apps. These insights help elucidate the socio-psychological behaviors of dating app users and can inform app design to reduce fatigue and enhance user well-being.

## 1. Introduction

Amidst the digital technology revolution, online dating applications have emerged as a predominant medium for initiating romantic connections and expanding social networks (Ferris & Duguay, 2020). Dating app usage has surged over the past decade, with the global user base of such apps reaching over 366 million in 2022 and projected to rise to 440 million by 2027 (Statista, 2023). Tinder—launched in 2012—stands out with its user-friendly design and innovative “swipe” feature, facilitating social and romantic explorations based on mutual interests and geographic proximity (David & Cambre, 2016; Statista, 2023).

However, the proliferation of dating apps has also led to “usage fatigue” among users, which has attracted scholarly attention (Dhir et al., 2018). Studies suggest that about one-third of users experience such fatigue, which reduces their engagement and satisfaction (Ranzini & Lutz, 2017; Ward, 2017). This fatigue is partly attributable to the rapid, appearance-based evaluation models used by these apps, which negatively impact self-perception and increase social anxiety (Ward, 2017).

Moreover, the FOMO is prevalent among users, driving frequent app checking to avoid missing out on potential social or romantic opportunities. This behavior traps users in a cycle of relentless usage, thus exacerbating anxiety and dissatisfaction as well as contributing to usage fatigue (Przybylski et al., 2013; Fu et al., 2023). Privacy concerns further compound this fatigue, as users must balance attracting potential partners and protecting their personal information (Fox & Warber, 2015).

This research employs the Stressor–Strain–Outcome (SSO) theoretical framework to elucidate the dynamics of the FOMO within dating app usage and its role in precipitating usage fatigue. In particular, it examines intermediary variables such as self-disclosure and social media stalking, which culminate in the JOMO experience. Privacy concerns are considered to be a moderating variable that influences the interplay between the FOMO and usage fatigue, ultimately impacting the perception of the JOMO (Chen & Lee, 2013).

Under this framework, the current research aims to bridge the gap in scholarly discourse regarding the transition from the FOMO to the JOMO among dating app users, thereby laying the theoretical and empirical groundwork necessary to enhance dating app design and user experience. The present study seeks to unravel the psychological evolution by which dating app users transition from the FOMO to the JOMO. It aims to explore strategies that might alleviate the FOMO’s negative repercussions on users and foster a positive shift in user experience through refined app design. This inquiry is pivotal in augmenting users’ social well-being and has significant implications for developers striving to address the psychological aspects of user engagement in the digital era.

## 2. Theoretical Background

### 2.1. The SSO Framework

The Stressor–Strain–Outcome (SSO) framework, which was developed by G.F. Koeske and R.D. Koeske in 1993 (Koeske & Koeske, 1993), categorizes human stress into stressors (stimuli provoking stress), strains (psychological responses), and outcomes (consequences of sustained stress). This model has been pivotal in studying social media’s impact on users, particularly by highlighting usability issues, privacy concerns, and negative perceptions as factors that increase usage fatigue (Valkenburg et al., 2022). Recent research applying the SSO model to platforms like Facebook, WeChat, and WhatsApp shows that stressors such as information overload and compulsive usage exacerbate user fatigue (Zhang et al., 2022).

The present study uses the SSO framework to examine the impact of the FOMO on user fatigue and the subsequent experience of the JOMO in dating apps. The FOMO drives intense social media engagement to avoid missing out, leading to anxiety and depression (Przybylski et al., 2013) and potentially resulting in social media avoidance (Rozgonjuk et al., 2020). Self-disclosure, which is a reaction to the FOMO, involves sharing personal information for social feedback (Gonzales & Hancock, 2011), while social media stalking, which is another FOMO outcome, involves monitoring others’ activities to alleviate one’s own missing out anxiety (Buglass et al., 2017).

Social media fatigue is a key SSO that results from prolonged behaviors like social media stalking and coping with FOMO-related stress (Matthes et al., 2020). Meanwhile, the JOMO is a positive outcome that occurs when users consciously reduce their own social media usage to mitigate the negative impacts of overuse (Kümpel et al., 2015).

In summary, we propose that the FOMO, as a social system stressor, leads users to engage more in self-disclosure and social media stalking to alleviate anxiety. This behavior may cause information overload and trigger social media fatigue. As users recognize this fatigue with themselves, they may strive for the JOMO to restore balance and reduce stress by reducing their social media engagement. This study aims to bridge the gap in understanding the transition from the FOMO to the JOMO among dating app users and offer insights that can help enhance app design and user experience.

### 2.2. FOMO, Self-Disclosure, and Social Media Stalking

The FOMO is a psychological state where individuals feel compelled to stay connected to others’ activities due to a fear of missing beneficial experiences (Przybylski et al., 2013; Yang et al., 2024). This fear is intensified by dating apps that specifically highlight the potentially enjoyable experiences that one might miss (Park, 2022). Research shows that the FOMO motivates users to engage with dating apps to avoid missing potential romantic encounters or disrupting ongoing connections (Schmitz & Zillmann, 2016). Feeling high levels of the FOMO causes users to seek more social connections to strengthen ties (Przybylski et al., 2013), suggesting that they may engage in self-disclosure to enhance and maintain relationships (Kroll & Stieglitz, 2021).

Self-disclosure—i.e., the act of sharing personal information with others (Collins & Miller, 1994)—is coming to be considered increasingly relevant in research examining social media interactions and privacy concerns (Nabity-Grover et al., 2020). On social media, self-disclosure includes posting images, personal details, and status updates (Pang et al., 2024). On dating apps like Tinder, it begins with profile creation and intensifies after mutual “right-swipes”, leading to deeper personal exchanges (Lee, 2019; Ward, 2016).

Research shows that online interactions often elicit greater self-disclosure than face-to-face communications (Knop et al., 2016), which enhances interpersonal relationships (Sprecher & Hendrick, 2004). In dating apps, self-disclosure is crucial for establishing and nurturing romantic connections (Ward, 2016). Studies have shown that there is a positive correlation between the FOMO and the propensity for self-disclosure (Talwar et al., 2019), suggesting that users might divulge more personal information in the pursuit of seizing social opportunities (Hann et al., 2007).

In summary, the FOMO on romantic opportunities or damaging one’s existing connections may trigger the FOMO, leading users to engage in self-disclosure activities to maintain or enhance relationships on dating apps. Thus, we propose the following:

**H1.**  *The FOMO positively influences the propensity of dating app users to engage in self-disclosure activities*.

Social media stalking refers to monitoring others’ profiles and shared content on social platforms, often as a benign form of cyberstalking (Dhir et al., 2021). For example, one might monitor an ex-partner’s life on Facebook to see if the ex-partner has any new romantic connections (Lyndon et al., 2011). This behavior involves gathering extensive information about others’ lives and is driven by voyeuristic tendencies (Mäntymäki & Islam, 2016). Meanwhile, on Tinder, user stalking behavior involves observing a potential partner’s social profiles and content shared on the platform.

Research indicates that users with a heightened FOMO are more inclined to use Facebook to monitor others (Park, 2022) to alleviate FOMO-associated anxiety. This suggests that users with the FOMO are likely to pay more attention to others’ social media dynamics (Milyavskaya et al., 2018; Tandon et al., 2021) and spend considerable time observing others’ profiles and activities (Tandon et al., 2021). Elhai et al. (2016) found a positive correlation between the FOMO and the frequency of social media use and stalking behaviors. Buglass et al. (2017) identified the FOMO as a primary driver for social media stalking, noting that users exhibiting a high FOMO engage in more frequent stalking to mitigate their anxiety of missing out on social information. Therefore, we propose the following:

**H2.**  *The FOMO positively influences dating app users’ propensity to engage in social media stalking behaviors*.

In summary, the FOMO drives users to disclose more personal information and engage in social media stalking to mitigate the anxiety of missing out on social opportunities, and this pathway is particularly visible in the context of dating apps. Although these behaviors help maintain and enhance romantic connections, they can also contribute to usage fatigue.

### 2.3. Usage Fatigue, FOMO, Self-Disclosure, and Social Media Stalking

Social media usage fatigue is a significant psychological phenomenon that is characterized by stress, exhaustion, and reduced vitality due to extended engagement (Ravindran et al., 2014; Ji & Jan, 2024). This fatigue is particularly prevalent among dating app users, where it stems from the demands of continuous interaction—such as swiping, matching, and conversation maintenance—on platforms like Tinder (Orosz et al., 2016). The exhaustion felt from such experiences often leads to temporary or permanent disengagement from these platforms (Solovyeva & Laskin, 2022).

The FOMO exacerbates this fatigue by driving users to prolonged social media use, which increases psychological distress, including depression and anxiety (Roberts & David, 2020). This cycle is particularly vicious in dating apps, where the quest for connection can foster negative emotional outcomes (Seo & Ray, 2019; Solovyeva & Laskin, 2022). In a comprehensive investigation of this topic, Coduto et al. (2020) highlighted how FOMO-driven usage creates a counterproductive loop that accelerates fatigue onset.

The FOMO significantly influences social media behaviors, in turn affecting usage frequency and post-use psychological states (Orosz et al., 2016). In online dating, the FOMO can heighten one’s app activity based on a desire to avoid missing potential matches, but this increased engagement may also fast-track fatigue symptoms (Coduto et al., 2020; Milyavskaya et al., 2018). Therefore, we propose the following:

**H3.**  *The FOMO positively influences the prevalence of usage fatigue among dating app users*.

Self-disclosure is pivotal for cultivating intimacy and social satisfaction in social media and dating app environments (Sprecher & Hendrick, 2004). Users engage in self-disclosure under the motivations of fostering long-term relationships, enhancing mutual understanding, and intimacy (Ellison et al., 2006). On platforms like Tinder, post-match communication via self-disclosure is essential for developing and reinforcing connections (Ward, 2016). While self-disclosure facilitates social interaction, it can also lead to usage fatigue, as the obligation to respond to extensive interactions imposes psychological pressure (Kaur et al., 2021).

The perceived demands of continual self-disclosure and maintaining an online persona intensify psychological burdens, particularly when having to process feedback from others (Pang et al., 2024). On dating apps, users must curate their online image and experience rejection and judgments, which can potentially lead to persistent psychological stress (Ward, 2017). This stress can evolve into usage fatigue, ultimately resulting in diminished interest and reduced engagement with the app (Sawyer et al., 2018).

Self-disclosure aimed at forming intimate connections often involves revealing sensitive personal information, leaving users vulnerable to negative outcomes like cyberbullying, online harassment, and privacy breaches (Bazarova & Choi, 2014). Consequently, self-disclosure likely exerts stress on users in the form of usage fatigue (Malik et al., 2020). Therefore, we propose the following:

**H4.**  *Self-disclosure positively influences usage fatigue among dating app users*.

Social media stalking involves closely monitoring others’ activities and personal information, which consumes significant psychological and temporal resources (Kircaburun et al., 2019). Stemming from a desire to stay informed, this behavior can lead to social media overuse and fatigue (Dhir et al., 2018; Seo & Ray, 2019).

While it may be gratifying to frequently observe others’ profiles, it can also lead to information overload and fatigue (Dhir et al., 2021). This pattern is particularly relevant in the context of dating apps like Tinder, where constant swiping and profile viewing can lead to overuse and fatigue (Ward, 2017). Studies show that social media stalking diminishes well-being and increases mental stress (Dhir et al., 2021). Prolonged usage and heightened engagement among dating app users can lead to usage fatigue, which is closely associated with the continuous attention and information processing demands of social media stalking. Moreover, persistent engagement may culminate in emotional exhaustion, which is exacerbated by the rejection and uncertainty in seeking romantic partners (Hobbs et al., 2017). Therefore, we propose the following:

**H5.**  *Social media stalking positively influences dating app users’ experience of fatigue*.

In summary, the FOMO, self-disclosure, and social media stalking all make significant contributions to usage fatigue among dating app users. It is crucial to understand these dynamics to enhance user experience and app design.

### 2.4. Usage Fatigue and the JOMO

User fatigue in dating apps mirrors that observed on regular social media platforms. Prolonged swiping, unsuccessful matches, and superficial interactions can lead to emotional exhaustion and a lack of fulfillment (Ranzini & Lutz, 2017). Users who fail to forge meaningful connections may feel their time investment yields minimal reward, which can lead to emotional drain and boredom (Hobbs et al., 2017).

A positive response to this fatigue is the emergence of the JOMO, which is marked by a deliberate shift away from digital consumption and toward embracing real-world experiences and interactions. This mindset shift acknowledges the negative impacts of excessive social media use, promoting mental well-being and life satisfaction by prioritizing offline activities (Kümpel et al., 2015). In dating apps, the move toward the JOMO can be a strategic retreat from the overwhelming demands of constant connectivity, allowing users to reclaim personal time and mental space for more fulfilling pursuits.

The transition from usage fatigue to the JOMO can be considered a natural coping mechanism for digital overload, where users consciously reduce their online presence to enhance their real-life engagements and relationships (Barry et al., 2023). This shift addresses the immediate stress and dissatisfaction associated with persistent app usage and fosters a healthier balance between digital and personal life, potentially improving mental health and quality of life (Dhir et al., 2018; Tromholt, 2016). Therefore, we propose the following:

**H6.**  *Usage fatigue positively influences dating app users’ experience of the JOMO*.

### 2.5. Mediation Effect

The transition from the FOMO to usage fatigue to the JOMO involves a gradual move from relentless social media engagement to valuing one’s personal time and space.

The FOMO drives intensified social media engagement and serves as an emotional stressor, which can potentially lead to user fatigue. Research by Przybylski et al. (2013) shows that, although the FOMO fosters increased social media activity, it can also precipitate user fatigue after prolonged use. Continuous information consumption and social interaction lead to cognitive and emotional strain, evolving into usage fatigue over time. This fatigue often encourages users to seek the JOMO by reducing their social media usage in pursuit of real-life contentment and serenity (Dhir et al., 2018). Therefore, we propose the following:

**H7.**  *Usage fatigue mediates the relationship between the FOMO and the JOMO, with the FOMO intensifying usage fatigue, which in turn fosters the JOMO*.

Self-disclosure refers to the active sharing of personal information with others in order to foster social connections and reduce feelings of loneliness. This behavior is typically associated with increased social engagement and can enhance one’s sense of belonging. Specifically, self-disclosure is a behavior wherein one aims to counteract the FOMO by sharing personal information to sustain social connections and alleviate anxiety over potential missed opportunities (Park, 2022). Extended self-disclosure can increase cognitive and affective burdens, thus leading to usage fatigue. When facing constant stress and fatigue, users may lean toward the JOMO with the aim of finding satisfaction beyond social media by reducing their social media usage and rediscovering the joy of real-life experiences (Tromholt, 2016; Dhir et al., 2018). Therefore, we propose the following:

**H8.**  *The FOMO amplifies usage fatigue through heightened self-disclosure, facilitating the JOMO experience*.

In contrast, social media stalking involves the passive observation of others’ social media activities to alleviate the FOMO or anxiety. While both behaviors are related to fatigue, self-disclosure typically aims to enhance social interaction, whereas social media stalking serves as a coping mechanism to reduce feelings of missing out, without the intention of direct social engagement. Specifically, social media stalking, driven by the FOMO, leads to overexposure to content, resulting in information overload, emotional exhaustion, and usage fatigue (Dhir et al., 2018). This behavior can eventually propel users toward the JOMO as a self-protective response, where they reduce online stress in favor of offline relaxation (Tandoc et al., 2015). Therefore, we propose the following:

**H9.**  *The FOMO exacerbates usage fatigue and subsequently fosters the JOMO through increased social media stalking*.

### 2.6. Moderating Variable: Privacy Concerns

Privacy concerns involve users’ worries about the management and use of their personal information on social media platforms (Smith et al., 2011). These concerns can heighten stress related to sharing personal information, thus increasing usage fatigue as users become more guarded about online disclosures (Smith et al., 2011). Feeling the need to take such cautious approaches can amplify stress when balancing the decision to share or withhold information, ultimately leading to greater cognitive and emotional strain.

Privacy concerns also modify the relationship between the FOMO and usage fatigue. While privacy worries might prompt users to exercise caution when perusing social media updates (Obar & Oeldorf-Hirsch, 2020), this prudence can heighten psychological stress. Reduced social media engagement due to privacy concerns may amplify the sense of missing out, reinforcing the FOMO and escalating usage fatigue (Buglass et al., 2017).

In social media stalking, users with pronounced privacy concerns might be more vigilant about their online actions, with this increased vigilance causing emotional weariness and psychological strain (Marshall et al., 2015). This vigilance can also increase usage fatigue as users frequently contemplate and self-regulate their behavior (Kaplan & Berman, 2010). Therefore, we propose the following:

**H10a–c.**  *Privacy concerns moderate the relationships between (a) self-disclosure, (b) the FOMO, and (c) social media stalking behaviors, thus impacting social media usage fatigue*.

In summary, privacy concerns reshape online interactions and can intensify user fatigue across self-disclosure, FOMO experiences, and social media stalking behaviors.

## 3. Research Methodology

### 3.1. Research Model

**Figure 1:**
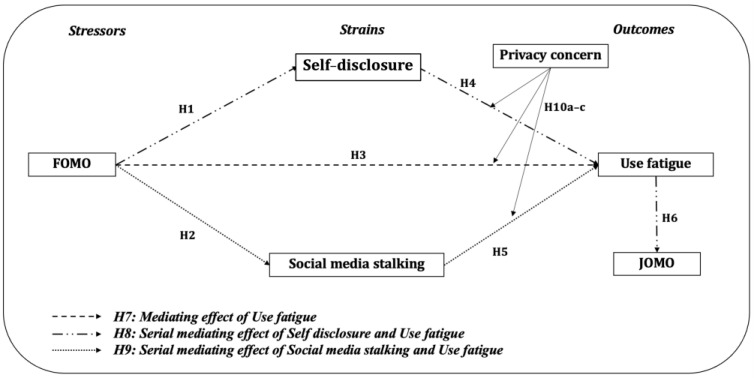
Research model.

Using the Stressor–Strain–Outcome (SSO) theoretical framework, this study scrutinizes the psychological transitions of dating app users from the “FOMO” to the “JOMO”. In essence, the proposed research model is as follows (Figure 1):

### 3.2. Study Measures

We adapted an existing scale for this study, as can be seen in Table 1. The “Privacy Concerns” scale, which is based on Buchanan et al. (2007) and Malhotra et al. (2004), includes items like, “I am concerned about my personal information (e.g., photo, name) being disclosed on a dating app”, all of which are rated on a seven-point Likert scale (1 = strongly disagree, 7 = strongly agree). Four experts in media psychology assessed the content validity, and the scale was refined based on their recommendations. A preliminary test with 20 Tinder users was conducted to ensure item clarity, as shown in Table 1.

### 3.3. Data Collection

The study sample was recruited over a 14-day period in October 2023 via the Chinese professional survey platform Questionnaire Star (www.sojump.com). Informed written consent was obtained from all participants prior to data collection. After conducting a data validity test, 410 valid samples were ultimately selected for analysis.

### 3.4. Participant Profiles

Most participants (70.98%) were aged 20–40 years, a demographic actively using dating apps for both short- and long-term relationships (Smith & Anderson, 2018). This age group, often with stable careers and social networks, uses dating apps as a convenient tool to form new connections (Slater, 2013). Furthermore, 86.83% of participants held at least a bachelor’s degree, indicating a digitally proficient and socially active user group. Gender distribution was relatively balanced (54.88% male, 45.12% female), offering insights into dating app use across different demographics, as shown in Table 2.

### 3.5. Data Analysis

Data analysis involved two phases: assessing validity and reliability through Confirmatory Factor Analysis (CFA) (Henseler et al., 2015), and examining hypotheses via structural equation modeling (SEM) using AMOS 27. SPSS 27 was employed for descriptive, correlational analyses, as well as to conduct tests for common method bias, mediation, and moderation.

## 4. Results

### 4.1. Data Normality and Common Method Bias

The skewness and kurtosis values were found to be within ±1.96, indicating normal distribution. The VIF values were below 5, and tolerance exceeded 0.10, indicating minimal multicollinearity. PCA on 20 variables yielded six components, accounting for 70.012% of variance, thus confirming a reliable factor structure.

### 4.2. Measurement Model: Reliability and Validity

Model fit indices: χ2/df = 1.905, CFI = 0.957, TLI = 0.953, RMSEA = 0.047. AVE and CR met the relevant benchmarks (AVE ≥ 0.5, CR ≥ 0.7). HTMT analysis confirmed discriminant validity with correlations below 0.85 (Henseler et al., 2015), affirming the distinctiveness of the constructs, as shown in Table 3, Table 4 and Table 5.

### 4.3. Structural Model

After the Confirmatory Factor Analysis (CFA), a path analysis was conducted to evaluate the overall model fit and the validity of the proposed hypotheses. The hypothesis testing results indicated that the model exhibited a robust fit, as evidenced by the benchmark fit indices (χ^2^/df = 1.905, Comparative Fit Index [CFI] = 0.957, Tucker–Lewis Index [TLI] = 0.953, Root Mean Square Error of Approximation [RMSEA] = 0.047). The path coefficients, which are presented in Table 6, further corroborate the six hypotheses H1 (β = 0.194 ***), H2 (β = 0.425 ***), H3 (β = 0.469 ***), H4 (β = 0.229 ***), H5 (β = 0.14 ***), and H6 (β = 0.304 ***), as shown in Table 6.

### 4.4. FOMO -> JOMO Multiple Mediation Analysis

Using PROCESS Model 4 with 5000 bootstrap samples, this study examined how self-disclosure and social media stalking mediate the link between FOMO-usage fatigue (UF), eventually leading to the JOMO (Table 7).

Path 1’s indirect effect (Ind2) was found to be 0.1074 (95% CI: 0.0379 to 0.1818), indicating significant mediation by UF, thus supporting H7. Path 2’s Ind2 showed an indirect effect of 0.1297 (95% CI: 0.0511 to 0.2093), also confirming UF’s mediation.

The indirect effects for Ind1 (0.0240, 95% CI: 0.0007 to 0.0605), Ind2 (0.1074, 95% CI: 0.0379 to 0.1818), and Ind3 (0.0085, 95% CI: 0.0006 to 0.0224) confirmed mediation by self-disclosure, thus supporting H8.

Ind1’s indirect effect (−0.0197, 95% CI: −0.0687 to 0.0303) indicated that there was no significant mediation by social media stalking (SMS), but Ind2 (0.1297, 95% CI: 0.0511 to 0.2093) and Ind3 (0.0132, 95% CI: 0.0007 to 0.0348) showed significant mediation by UF and SMS, respectively, partially supporting H9, as shown in Table 7.

### 4.5. Moderating Effect of Privacy Concerns

**Figure 2:**
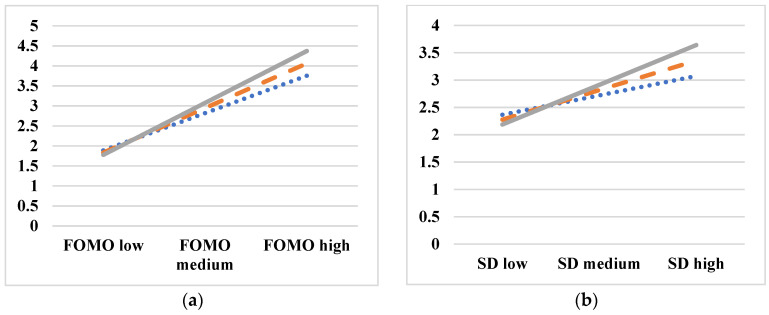
(**a**) Moderating effect of privacy concerns on the association between the FOMO and UF; and (**b**) moderating effect of privacy concerns on the association between SD and UF. The gray line represents the high group, the orange line represents the medium group, and the blue dotted line represents the low group.

The results of moderation analyses using Model 1 in the PROCESS macro with 5000 bootstrapping iterations revealed that privacy concerns significantly moderated the effects of usage fatigue related to the FOMO and self-disclosure, as shown in Table 8 and Figure 2. Further, higher privacy concerns intensified the positive relationships between both self-disclosure and the FOMO with usage fatigue. However, privacy concerns did not significantly affect the relationship between social media stalking and usage fatigue. Thus, Hypotheses 10a and 10b are supported, whereas Hypothesis 10c is not supported.

## 5. Discussion

This study explores the transition from the FOMO to the JOMO and examines how privacy concerns influence this shift. The validation of hypotheses H1, H2, and H3 demonstrate the significant impact that the FOMO has on the behaviors of dating app users. H1 reveals that the FOMO positively influences self-disclosure behaviors, thus enhancing one’s appeal and increasing one’s chances for social engagement and successful pairings (Ward, 2017). This underscores the crucial role played by the FOMO in digital social interactions within dating apps, which are characterized by immediacy and prospective connections. H2 indicates that the FOMO increases users’ propensity for social media stalking, driven by concerns about potential rivals or a desire to manage their social environment. H3 shows a positive correlation between the FOMO and usage fatigue, suggesting that excessive reliance on dating apps due to the FOMO can lead to psychological and emotional exhaustion (Elhai et al., 2016), negatively affecting users’ engagement with the app and their well-being.

The data robustly support both hypotheses H4 and H5, respectively, demonstrating that self-disclosure and social media stalking on dating apps significantly contribute to usage fatigue. While self-disclosure is essential for forming connections and attracting partners, excessive self-presentation efforts can induce psychological strain (Ward, 2017). Similarly, the relentless scrutiny of others’ activities amplifies users’ psychological burden (Tandoc et al., 2015). These findings highlight the importance of managing self-disclosure and social media stalking in digital dating to mitigate usage fatigue and preserve psychological well-being.

Hypothesis H6 confirms that Tinder usage fatigue constructively contributes to the JOMO. As users become overwhelmed by continuous interactions, they reduce their use of Tinder as an aspect of adopting a more mindful approach to digital media and reallocating their time to fulfilling offline activities. This shift leads to greater satisfaction in real-world engagements and improved social bonds. Reducing one’s online engagement has been shown to enhance life quality and strengthen social relationships (Shensa et al., 2017). Further, distancing from social media is associated with improved concentration and fewer mental distractions, thus linking the JOMO with better attention and presence (Panova & Lleras, 2016). Our research highlights the role played by Tinder usage fatigue in encouraging real-life interactions, specifically by promoting a healthier digital and social lifestyle.

The present study has delved into how the FOMO leads to usage fatigue and the JOMO through psychological and behavioral pathways, including self-disclosure and social media stalking. The results supporting Hypothesis H7 confirm that usage fatigue (UF) mediates the transition from the FOMO to the JOMO. This suggests a psychological adaptation in which individuals, overwhelmed by the FOMO, reduce their social media engagement to alleviate stress. This reduction promotes a positive emotional state towards social media use, referred to as the JOMO. This finding is consistent with Elhai et al. (2016), who observed that continuous online engagement can lead to usage fatigue, driving users towards the JOMO—the experience of contentment outside the digital realm. Our study demonstrates how the FOMO leads to usage fatigue and facilitates the transition to the JOMO through psychological and behavioral pathways such as self-disclosure and social media stalking. By confirming Hypothesis H7, we emphasize usage fatigue as a key mediator. This shows that individuals adapt by reducing online activity, thereby achieving well-being through decreased social media use, which in turn supports the JOMO experience. Hypothesis H8 reveals that the FOMO drives users to increase self-disclosure on platforms like Tinder to connect and affirm their identity, but this leads to fatigue due to the emotional and cognitive investment required (Alt, 2015; Park, 2022). This fatigue encourages digital disengagement, ultimately resulting in the JOMO. The mediation process described in Hypothesis H9 shows that FOMO-induced social media stalking exacerbates usage fatigue, indirectly facilitating the JOMO. While stalking does not directly result in the JOMO, the resulting fatigue reduces online engagement, thereby enhancing real-life satisfaction (Lee et al., 2016).

Although Hypothesis 9 posits that social media stalking mediates the transition from the FOMO to the JOMO, the analysis reveals that the direct effect of social media stalking on the relationship between the FOMO and the JOMO is insignificant (β = −0.0197, *p* > 0.05), with only a marginal indirect effect (β = 0.0132, *p* < 0.05). This suggests that social media stalking may not play as significant a role as self-disclosure in mediating the transition from the FOMO to the JOMO. This could be attributed to the relatively indirect nature of social media stalking behavior, which may not be the primary driver of psychological adaptation, and may be influenced by other psychological factors or the characteristics of the platform. Our findings emphasize the need to balance digital engagement with offline interactions for psychological well-being, highlighting the mediating role that usage fatigue plays in the FOMO-to-JOMO transition. Although both self-disclosure and social media stalking are linked to the transition from the FOMO to the JOMO, they serve different psychological functions. Self-disclosure is an active coping mechanism that fosters social connection, while social media stalking is a passive activity aimed at alleviating anxiety related to missing out. Given these differences, it is important to consider each behavior separately when examining their effects on the psychological outcomes associated with social media use.

The emergence of privacy concerns within dating applications intensifies the stress associated with self-disclosure and the FOMO, eventually leading to increased usage fatigue and potentially catalyzing the pursuit of the JOMO. Hypotheses H10a and H10b confirm that privacy concerns amplify the link between self-disclosure and usage fatigue, underscoring the psychological strain stemming from concern about potential privacy breaches (Ward, 2017). Privacy concerns also moderate the FOMO’s impact on usage fatigue, highlighting how anxiety over potential matches exacerbates fatigue for users with privacy apprehensions (Sumter et al., 2017). However, Hypothesis H10c was not supported, indicating that there is no substantial moderating effect between social media stalking behavior and usage fatigue. This suggests the existence of a complex interplay between stalking behaviors, privacy concerns, and their impact on fatigue (Ranzini & Lutz, 2017). This study’s findings emphasize the importance of taking a balanced approach to online engagement and offline interactions for optimal well-being.

## 6. Theoretical and Practical Implications

### 6.1. Theoretical Implications

This study makes a significant contribution to the current theoretical understanding of dating app users’ behavior by applying the Stress–Strain–Outcome (SSO) framework to the transition from the FOMO to the JOMO.

First, the results of this study confirm that the FOMO intensifies self-disclosure and social media stalking (H1 and H2), supporting Przybylski et al.’s (2013) theory of the FOMO as a driver of online engagement. It extends this theory by demonstrating that the FOMO not only stimulates these behaviors but also leads to usage fatigue (H3), which eventually results in the JOMO (H7–H9). This shift reflects a psychological adaptation toward self-regulation (H6), which aligns with Burke et al.’s (2010) exploration of digital anxiety and disengagement.

Secondly, these findings highlight the adverse psychological effects of prolonged digital interaction. These results show that both self-disclosure (H4) and social media stalking (H5) contribute to usage fatigue, aligning with the existing literature reporting on the negative consequences of sustained social media activity (Dhir et al., 2018). These findings underscore the importance of balancing one’s social media use to maintain mental health and psychological boundaries.

Thirdly, this study provides significant insights into the role played by privacy concerns as a moderating variable (H10a-c). It illustrates how these concerns influence the relationship between the FOMO, self-disclosure, and user fatigue. This supports the Privacy Management Theory by Masur (2020), thus emphasizing the delicate balance that users must strike between sharing personal information and maintaining privacy.

### 6.2. Practical Implications

First, this study highlights FOMO’s role in promoting self-disclosure and social media stalking on dating apps. Users should be aware of the psychological risks of overuse and adopt strategies such as setting usage limits, engaging in offline activities, and using app time management features to mitigate fatigue and foster the JOMO (Hook, 2023).

Secondly, privacy considerations are crucial. Developers should enhance privacy protection by providing users with more granular options and prompting them about potential risks when sharing personal information, ultimately allowing for safer social engagement.

Thirdly, dating app companies should prioritize users’ long-term well-being over short-term engagement. This includes striving to foster genuine relationships and meaningful communication, optimizing matching algorithms to align with users’ interests, and integrating mental health resources and usage recommendations to promote sustainable use patterns (Alt & Boniel-Nissim, 2018).

Lastly, platforms must responsibly regulate user content to prevent exacerbating the FOMO or social anxiety. It is essential for such platforms to implement stringent content moderation policies and provide support to users in distress due to overuse to promote healthy interactions. For example, platforms could combine algorithms with human moderation to filter content that may induce the FOMO or social anxiety. Additionally, platforms might offer psychological support services, such as counseling and emotional support, to help users manage distress and alleviate social anxiety.

## 7. Limitations and Future Research Directions

The current study has several limitations that should be noted. First, the findings are primarily applicable to dating apps such as Tinder, where self-disclosure and social media monitoring are common. These findings may not be directly applicable to other social media platforms, such as Facebook or Instagram, where user behavior might be driven by different motivations. For example, the FOMO in dating apps may exert a stronger influence on self-disclosure and social media monitoring due to the nature of user interactions, while privacy concerns and social image management might be more prominent on other platforms. Consequently, further research is needed to determine whether these findings are applicable across different social media platforms.

Second, a limitation of this study is the inability to conduct group comparisons based on gender, age, and education level. This limitation arises from factors such as sample size constraints, uneven distribution across demographic groups, and privacy concerns. Future research could overcome this by incorporating larger, more diverse samples and exploring how gender, age, and education level may influence users’ experiences of the FOMO and the JOMO.

Third, the cross-sectional design limits our understanding of how psychological states change over time. While this study identifies relationships between the FOMO, self-disclosure, and social media stalking, it cannot track temporal changes. Longitudinal studies are necessary to explore how the FOMO and the JOMO evolve and to clarify the causal relationships between these variables.

Fourth, regarding the results of Hypothesis 9, although social media stalking plays some role in the transition from the FOMO to the JOMO, its effect was marginal, with only a weak indirect effect and no significant direct effect. This suggests that social media stalking may not be as influential in this transition as initially hypothesized. Future research should further explore the relative importance of self-disclosure and social media stalking in the FOMO-to-the-JOMO transition, particularly across different platforms and social interaction contexts, to gain a deeper understanding of their roles.

Lastly, treating privacy concerns as a single moderating variable oversimplifies their complexity. Privacy concerns vary among users with different backgrounds and expectations. Future research should decompose privacy concerns into specific dimensions, such as informational control, trust in privacy policies, and perceived risks associated with sharing personal data. This approach will offer a more nuanced understanding of their role in the relationship between the FOMO and the JOMO.

## Figures and Tables

**Table 1:** Study measures and factor loadings for the measurement and structural models.

Study Measures	Measurement Items	CFA	SEM
FOMO (Przybylski et al., 2013; Alt, 2015)	F1: I constantly check dating apps to see what my potential dates are doing, even on holidays.	0.817	0.817
	F2: I worry that my potential date is having fun without me.	0.774	0.774
	F3: I get anxious about not hearing from potential dates.	0.824	0.824
	F4: I feel anxious about not being able to attend a date with a potential date.	0.791	0.791
	F5: I worry that a potential date is more popular in the dating app than I am.	0.756	0.756
	F6: I frequently visit the dating app for new information.	0.791	0.791
	F7: I think it’s important to post recently trending topics and information on the dating app (e.g., videos, photos, etc.).	0.798	0.798
	F8: I get anxious about not responding to updates on the dating app.	0.760	0.760
	F9: I constantly check my phone to make sure I don’t miss any new messages from the dating app.	0.742	0.742
	F10: I share on the dating app when I’m having fun (e.g., personal status messages, daily life updates, etc.).	0.729	0.729
	F11: I think it’s important to know what jokes, buzzwords, or slang a potential date might say.	0.761	0.761
Self-discourse (Wheeless & Grotz, 1976)	SD1: I take the time to update my profile and related dynamics on the dating app.	0.858	0.858
	SD2: I reveal a lot of personal information about myself in my dating app profile.	0.810	0.810
	SD3: I am willing to disclose a lot of personal information about myself (e.g., my name, educational background, job, etc.) on a dating app for a potential date to get to know me.	0.773	0.773
	SD4: I enjoy sharing my feelings and daily life updates on dating apps.	0.870	0.870
	SD5: When I want to express something, I share it on a dating app.	0.723	0.723
Social media stalking (Dhir et al., 2021; Smoker & March, 2017)	SMS1: I check the profiles of potential dates on dating apps, snooping on their personal information, life events, etc.	0.854	0.854
	SMS2: I monitor potential dates on a dating app by looking at their posted profiles and updates.	0.813	0.813
	SMS3: I spend a lot of time on dating apps checking out the profiles and dynamics of potential dates.	0.828	0.828
	SMS4: I check out the updates posted by potential dates on the dating app to get a detailed picture of their daily activities (e.g., who they went where with on a particular day).	0.832	0.832
	SMS5: I look at a potential date’s personal information and related dynamics on a dating app to get a feel for their other emotional relationships (love, friendship, intimacy, etc.).	0.822	0.822
Use fatigue (Dhir et al., 2018; Whelan et al., 2020)	UF1: I feel mentally exhausted (e.g., empty, bored, or lonely) from using dating apps.	0.860	0.860
	UF2: I get so tired of using the dating app that I can’t do other tasks well.	0.804	0.804
	UF3: I feel very tired and fatigued after using the dating app.	0.817	0.817
	UF4: I have a hard time relaxing after using a dating app for an extended period.	0.852	0.852
	UF5: I get nervous because there are so many messages of all types on dating apps.	0.831	0.831
JOMO (Barry et al., 2023)	J1: I feel more relaxed and happier when I don’t use a dating app to connect with potential dates.	0.741	0.741
	J2: I don’t use dating apps when I’m on holiday.	0.786	0.786
	J3: I experience a feeling of peace when I take a short break from using dating apps.	0.806	0.806
	J4: I feel that deciding not to use a dating app or terminating communication with a potential date can help me regain balance in my life.	0.734	0.734

**Table 2:** Demographic information.

Category	Subcategory	Number of Participants	Percentage (%)
Gender	Male	225	54.88
	Female	185	45.12
Age Group	Under 20	43	10.49
	20–25	65	15.85
	25–30	103	25.12
	30–40	123	30
	40–50	56	13.66
	Over 50	20	4.88
Education Level	High School or below	41	10
	Bachelor’s Degree	243	59.27
	Master’s Degree	85	20.73
	Doctorate	28	6.83
	Other	13	3.17

**Table 3:** Goodness of fit for confirmatory factorial analysis (CFA).

CMIN/DF	RMR	GFI	AGFI	TLI	CFI	RMSEA
1.905	0.056	0.891	0.873	0.953	0.957	0.047
<3	<0.08	>0.85	>0.85	>0.9	>0.9	<0.08

**Table 4:** Results of validity and reliability analysis.

	CR	AVE	MSV	ASV	FOMO	SD	SMS	UF	JOMO
FOMO *	0.944	0.64	0.3283	0.153	0.8				
SD	0.904	0.72	0.1056	0.047	0.194	0.848			
SMS	0.917	0.751	0.1806	0.078	0.425	0.033	0.867		
UF	0.919	0.754	0.3283	0.162	0.573	0.325	0.347	0.868	
JOMO	0.851	0.69	0.0924	0.0528	0.256	0.209	0.099	0.304	0.831

* Fear of missing out (FOMO), social media stalking (SMS), self-disclosure (SD), use fatigue (UF), composite reliability (CR), average variance extracted (AVE), maximum shared variance (MSV), and average shared variance (ASV). Bold diagonal values represent respective square roots of AVE, while off-diagonal values represent inter-construct correlations.

**Table 5:** HTMT analysis.

	FOMO	JOMO	SD	SMS	UF
FOMO					
JOMO	0.284				
SD	0.205	0.237			
SMS	0.453	0.115	0.046		
UF	0.613	0.342	0.353	0.374	

**Table 6:** Results of hypotheses testing.

Hypothesis	Path	ß	Significance	Support
H1	FOMO → SD	0.194	<0.05	Yes
H2	FOMO → SMS	0.425	<0.001	Yes
H3	FOMO → UF	0.469	<0.001	Yes
H4	SD → UF	0.229	<0.001	Yes
H5	SMS → UF	0.14	<0.01	Yes
H6	UFSD → JOMO	0.304	<0.001	Yes

**Table 7:** Coefficients of the full multiple mediation model.

Mediation Path	Effect Value	Boot SE	95% Confidence Intervals	
			LLCI	ULCI
Path1-FOMO -> SD -> UF -> JOMO				
Direct effect of FOMO -> JOMO	0.1266	0.0594	0.0098	0.2434
Total indirect effect	0.14	0.0471	0.0537	0.2364
Ind1 (FOMO -> SD -> JOMO)	0.024	0.0158	0.0007	0.0605
Ind2 (FOMO -> UF -> JOMO)	0.1074	0.0367	0.0379	0.1818
Ind3 (FOMO -> SD -> UF -> JOMO)	0.0085	0.0058	0.0006	0.0224
Path2-FOMO -> SMS -> UF -> JOMO				
Direct effect of FOMO -> JOMO	0.1434	0.0625	0.0204	0.2663
Total indirect effect	0.1232	0.0496	0.0253	0.2219
Ind1 (FOMO -> SMS -> JOMO)	−0.0197	0.0248	−0.0687	0.0303
Ind2 (FOMO -> UF -> JOMO)	0.1297	0.0401	0.0511	0.2093
Ind3 (FOMO -> SMS -> UF -> JOMO)	0.0132	0.0089	0.0007	0.0348

**Table 8:** Results of moderation analysis.

	β	t	p	LLCI	ULCI	Moderation
H10a SD → UF	0.0931	2.7005	0.0072	0.0253	0.1608	Yes
H10b SMS → UF	−0.0071	−0.2236	0.8232	−0.0691	0.055	No
H10c FOMO → UF	0.0914	3.2	0.0015	0.0353	0.1476	Yes

## Data Availability

The data presented in this study are available upon request from the corresponding authors.
